# TransCluster: A Cell-Type Identification Method for single-cell RNA-Seq data using deep learning based on transformer

**DOI:** 10.3389/fgene.2022.1038919

**Published:** 2022-10-11

**Authors:** Tao Song, Huanhuan Dai, Shuang Wang, Gan Wang, Xudong Zhang, Ying Zhang, Linfang Jiao

**Affiliations:** ^1^ College of Computer Science and Technology, China University of Petroleum (East China), Qingdao, China; ^2^ Department of Artificial Intelligence, Faculty of Computer Science, Campus de Montegancedo, Polytechnical University of Madrid, Boadilla Del Monte, Madrid, Spain

**Keywords:** cell-type identification, single-cell sequencing data, transformer, neural network, deep learning

## Abstract

Recent advances in single-cell RNA sequencing (scRNA-seq) have accelerated the development of techniques to classify thousands of cells through transcriptome profiling. As more and more scRNA-seq data become available, supervised cell type classification methods using externally well-annotated source data become more popular than unsupervised clustering algorithms. However, accurate cellular annotation of single cell transcription data remains a significant challenge. Here, we propose a hybrid network structure called TransCluster, which uses linear discriminant analysis and a modified Transformer to enhance feature learning. It is a cell-type identification tool for single-cell transcriptomic maps. It shows high accuracy and robustness in many cell data sets of different human tissues. It is superior to other known methods in external test data set. To our knowledge, TransCluster is the first attempt to use Transformer for annotating cell types of scRNA-seq, which greatly improves the accuracy of cell-type identification.

## 1 Introduction

Recent advances in single-cell RNA sequencing (scRNA-seq) have furthered the understanding of cell compositions in complex tissues (Haque et al., 2017). Through the characterization of different cell types based on gene expression levels, facilitating our understanding on disease pathogeneses, cellular lineages or differentiation trajectories and cell-cell communication (Macosko et al., 2015; Regev et al., 2017; Potter, 2018; Shao et al., 2020). In the data processing protocols of scRNA-seq experiments, cell type identification is a key step in the subsequent analysis. The current strategies are divided into two main types, unsupervised-based and supervised-based annotation strategies. Unsupervised-based strategy applies clustering to classify cells into different clusters (Su et al., 2021; Tian et al., 2021). Several methods including Scanpy (Wolf et al., 2018), Seurat (Butler et al., 2018), SINCERA (Guo et al., 2015), SC3 (Kiselev et al., 2017), SIMLR (Wang et al., 2017), SNN-Clip (Xu and Su, 2015), BackSPIN (Zeisel et al., 2015) belong to this category. This type of approach requires *a priori* knowledge about known cellular markers. Replicability of this cell identification protocol can be further reduced with increased number of clusters and multiple selections of cluster marker genes (Shao et al., 2020; Wang et al., 2021). Supervised-based strategy determines potential cell identity by comparing similarities between individual cells and reference databases of bulk or scRNA-seq profiles, such as scDeepSort (Shao et al., 2021), SingleR (Aran et al., 2019), ACTINN (Ma and Pellegrini, 2020), singleCellNet (Tan and Cahan, 2019), scMap-cell (Kiselev et al., 2018). Still, the accurate cell type annotation for single-cell transcriptomic data remains a great challenge (Lahnemann et al., 2020).

Fortunately, recent advances in deep learning have enabled significant progress in the ability of artificial intelligence techniques to integrate big data, incorporate existing knowledge and learn arbitrarily complex relationships (Gibney, 2015; Silver et al., 2016). Given the state-of-the-art accuracy deep learning has achieved in numerous prediction tasks, it has been increasingly used in biological research (Zhang et al., 2019) and biomedical applications (Wainberg et al., 2018; Lv et al., 2020). For example, Jian Hu et al. proposed the ItClust method, which uses deep neural networks to learn feature expressions on the source data and then migrate to the target data to cluster the unknown labeled cells (Hu et al., 2020). One of the commonly used deep learning methods is convolutional neural networks (CNNs) (Qian et al., 2018), a class of feedforward neural networks. In CNNs, convolutional operations are good at extracting local features but have difficulties in capturing global representations. Since the introduction of Transformer (Vaswani et al., 2017), it has shown breakthrough performance in many learning tasks, and its strength lies in its ability to capture global contextual information in an attentional manner to establish a long-range dependence on the target (Song et al., 2022). We propose a new deep learning-based method for single-cell category prediction by combining CNN with Transformer to extract more powerful features.

In this study, we designed a cell type identification method called TransCluster, based on the Transformer framework. To the best of our knowledge, this is the first time that Transformer is applied to the field of single cell identification. Firstly, we prepared single-cell transcriptional profiles of different tissues in the human body (Han et al., 2020), as the training set. Next, we used the improved Transformer model for feature extraction of the gene expression matrix. Then, features were further extracted using CNN. Finally, we compared the performance of TransCluster with other known methods on an external dataset. In addition, we evaluated the performance of TransCluster with eight additional human tissue scRNA-seq atlases. The results demonstrated that TransCluster is a robust method that can help scientists achieve the accurate cell-type annotation of scRNA-seq data without additional prior knowledge.

## 2 Materials and methods

### 2.1 Datasets

The scRNA-seq data (Shao et al., 2021; Pang et al., 2022) were obtained from the Shao et al. (2021) and Baron et al. (Hu et al., 2020). The Shao dataset includes primary tissues from human and mouse, which exclude unannotated cells. The Baron dataset is a large human pancreas dataset. Human gene symbols were modified based on NCBI gene data, unmatched genes and duplicated genes were removed, and for all human datasets, the raw data were normalized by the global-scaling normalization method LogNormalize. On the one hand, we selected human tissues to verify the applicability of TransCluster on different tissues, including pancreas, human peripheral blood, adipose, adrenal gland, liver, kidney, spleen and pleura, with a total of 8 tissues and 51744 cells and the number of cell categories are 14, 10, 7, 9, 11, 7, 9, and 5 respectively. For each cell type, cells numbering at least more than 5‰ of the total cells in each tissue, the ratio of training and testing cells was set to 8:2, randomly divided into training and testing sets, and five experiments were performed, with the average value taken as the final result. On the other hand, to compare the accuracy of TransCluster with other methods, all cells from a particular tissue were used to train the model for cell-type prediction on the test cells that originated from the same tissue. Firstly, genes with zero expression in both datasets were removed to decrease the amount of data and to reduce the effect of irrelevant information (it was experimentally verified that different gene classes of cells in both datasets could still make accurate predictions). Secondly, cell types that were present in both datasets were selected to avoid unknown cell types. Finally, cells that were present in both datasets were removed to ensure the objectivity of the prediction results. The lung dataset has six categories of cells, including Transformed epithelium, AT2 cell, Macrophage, T cell, Endothelium and Fibroblast. In the kidney dataset, there are three categories of Endothelial cell, Epithelial cell, and Proximal tubule epithelial cell. In the blood dataset, there are four categories of B cell, Dendritic cell, Monocyte, and T cell. In addition, we have done a large number of stability experiments to determine the hyperparameters of the model and show the experimental results in the paper.

### 2.2 Model architecture

**FIGURE 1:**
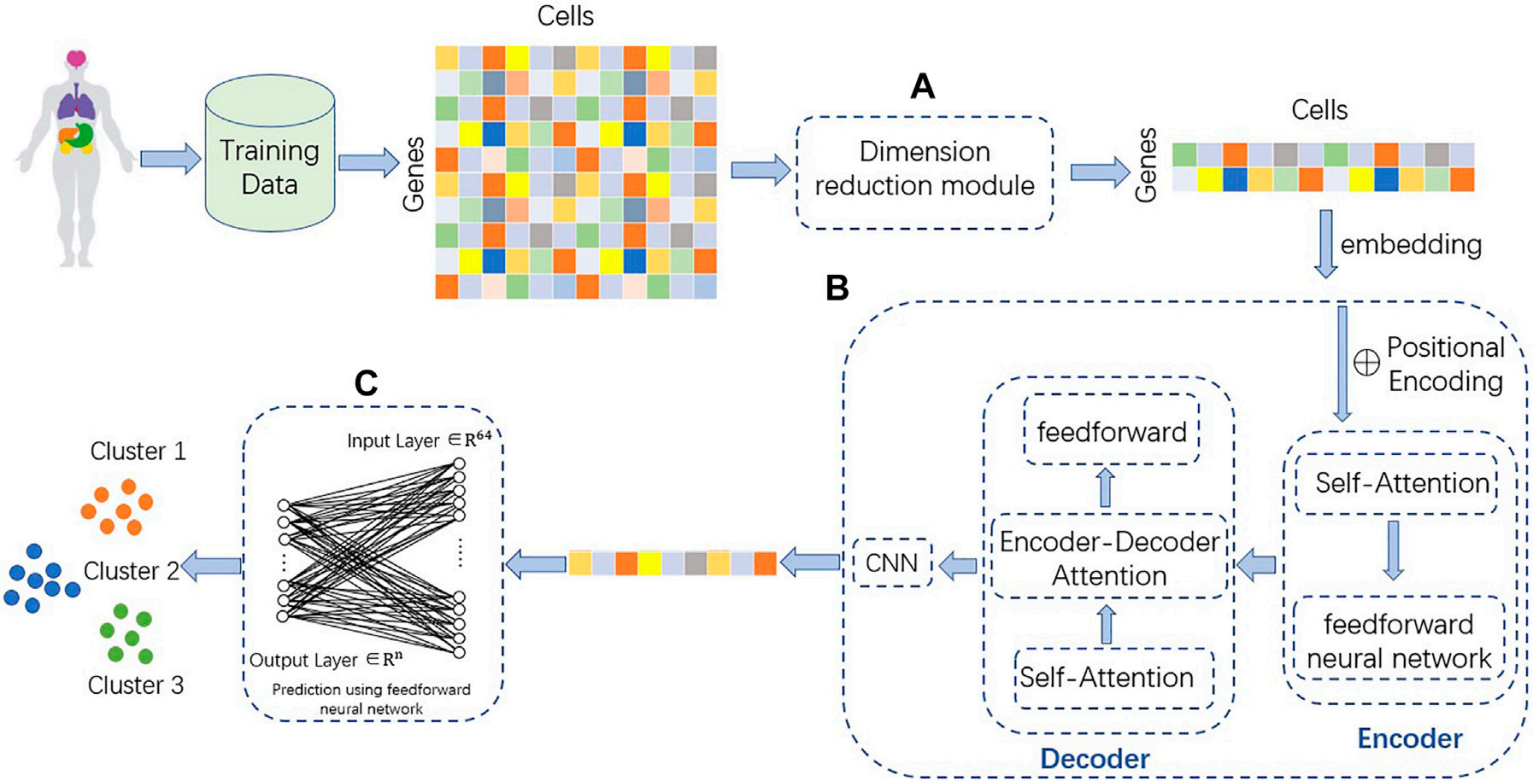
Structure of TransCluster. The input of TransCluster is the gene expression matrix of cells and the category of cells. **(A)** The cellular gene expression matrix was downscaled by the LDA method according to the cell category. **(B)** Feature extraction of the input processed gene expression matrix by **(A)** using a modified transformer and a one-dimensional CNN. **(C)** Classification of cells using linear classifiers, n is the number of categories.

TransCluster consists of three components: A dimensionality reduction part, a weighted feature extractor and a linear classifier. The dimensionality reduction component uses linear discriminant analysis (LDA) (Hastie et al., 2009), a supervised machine learning algorithm that reduces features to the appropriate dimension based on the labels of the data. The weighted feature extractor inductively learns the feature information of the cells and generates linear separable feature space of the cells. In this layer, a modified version of the Transformer (Vaswani et al., 2017) is used as the backbone, combined with a one-dimensional CNN (Qian et al., 2018). The final linear classifier classifies the final cell state representation generated from the weighted feature extractor into one of the predefined cell type categories. The structure of the model is shown in Figure 1.

#### 2.2.1 Linear discriminant analysis

As shown in Figure 1, we use linear discriminant analysis (LDA) to reduce the dimensionality of the gene expression matrix. In order to solve a multilabel classification problem efficiently and effectively, we need not only to consider the correlation of class labels and features of each data item but also to take into account the different cardinalities of the classes (Xu et al., 2021). The basic idea of LDA is to project the high-dimensional samples into the optimal discriminant vector space in order to extract the categorical information and compress the spatial dimensionality (Guo et al., 2020). At the same time, the projection ensures that the samples have the maximum inter-class distance and the minimum intra-class distance in the new subspace, i.e., the samples have the best separability in this space (Xu et al., 2021). For the input single-cell data matrix (the number of genes is m, the number of cells is n, the number of classes is k and the dimension after dimensionality reduction is d), it is experimentally verified that the best performance is achieved when d equals k-1. So, the dense representation with fixed size dimension k-1is extracted as the initial representation. The matrix after LDA processing is transposed, each number in the matrix is added with the same number so that all matrix numbers are positive, and table headers are added to obtain the reduced-dimensional gene expression matrix.

$$D^{\prime }={\left[LDA\left(D\right)\right]}^{T}+A$$ (1)

where D is the gene expression matrix input to the LDA module, A is a suitable positive matrix with each element being an identical positive number, and D′ is the output matrix after partial processing by dimensionality reduction.

#### 2.2.2 Weighted feature extractor

Transformer uses multi-head attention instead of recurrent layer or convolutional layer to extract information, which improves the performance of multiple tasks in natural language processing (NLP) (Vaswani et al., 2017; Sun et al., 2021). Compared with convolutional neural network (CNN) (Qian et al., 2018) and recurrent neural network (RNN) (Hochreiter, 1998), Transformer shows superior ability to deal with long-range dependencies (Guo et al., 2020). Multi-head attention mechanism enables Transformer to learn the features of different subsequences in the sequence (Wang et al., 2022). Transformer is capable of linking different positions of a sequence to obtain an embedding containing contextual information when processing sequence information (Baron et al., 2016).

The LDA-encoded sequence is input to Transformer to generate a feature vector $T_{transformer}$ , which contains sequence structure information.

$$T_{transformer}=Transformer\left(D^{\prime }\right)$$ (2)

Where D′ is the output matrix after partial processing by dimensionality reduction, $T_{transformer}$ is the gene expression matrix after transformer processing.

Self-attention layer, Firstly, each gene expression in the LDA reduced matrix is considered as a vector, and Transformer multiplies each vector of the input by three matrices to obtain three new vectors Q, K, and V, thus adding more parameters and improving the model effect. The attention score is calculated by computing the dot product of Q and the K vector of each gene. The obtained scores are normalized with SoftMax. The V-vector of each gene is multiplied by the normalized value to the output of the self-attentive layer, with the following equation.

$$Attention\left(Q,K,V\right)=SoftMax\left(\frac{QK^{T}}{\sqrt{d_{k}}}\right)V$$ (3)

Where Q is the query vector, K denotes the vector of relevance of the queried information to other information, V denotes the vector of queried information, and $d_{k}$ is the dimension of the key vector.

Multi-Head Attention, Each head computes its own Attention, and then multiplies it to obtain the final feature representation after stitching.

$$MultiHead\left(Q,K,V\right)=Concat\left(head_{1},...,head_{h}\right)W^{o}$$ (4)

Where $head_{i}=Attention\left(Q,K,V\right)$ , Concat is a bitwise sum operation, $W_{i}^{o}\in R^{hd_{v}\times d_{model}}$ , which is the weight matrix. If h=8 , then $d_{k}=d_{v}=d_{model/h}=64$ , Setting different h = {1,2,3,4,5,6,7,8,9,10} to do sensitivity tests, the results show that the model works best with h = 5. Therefore, our model takes head = 5.

Position-wise Feed-Forward Networks, The position fully connected feed-forward network has two dense layers, the first layer has a Relu activation function and the second layer is a linear activation function. Position-wise means that the input and output dimensions are the same. The formulation is stated below:

$$FFN\left(x\right)=\max{\left(0,xW_{1}+b_{1}\right)W}_{2}+b_{2}$$ (5)

Where x denotes the multi-head output. $xW_{1}+b_{1}$ denotes a linear transformation, max represents the Relu activation function, and $W_{2}$ and $b_{2}$ are the weights of the second linear transformation.

Positional encodings, Cos and sin functions are used to encode the position and enhance the model’s ability to perceive the position information. The formulas are as follows.

$${PE}_{\left(pos,2i+1\right)}=\sin\left(\frac{pos}{10000^{2i/d_{model}}}\right)$$ (6)

$${PE}_{\left(pos,2i+1\right)}=\cos\left(\frac{pos}{10000^{2i/d_{model}}}\right)$$ (7)

Where pos indicates the position of the gene, i indicates the dimension of the gene, $d_{model}$ denotes the dimension of embedding.

The Transformer-processed matrix $T_{transformer}$ is fed into the 1D-CNN network (Han et al., 2020) for further feature extraction. Where T is the sequence after one-dimensional convolutional processing.

$$T=CNN\left(T_{transformer}\right)$$ (8)

#### 2.2.3 Linear classifier layer

The features extracted by the CNN are fed into a linear classifier for category prediction (Baron et al., 2016) and the probabilities of each category are output. The features go through nonlinear changes in the dense layer to extract the association between these features and finally map them to the output space. The activation function is softmax. The loss function is calculated as follows. Where $y_{i}$ is the real label and $\hat{y_{i}}$ is the predicted label.

$$Loss=-\overset{output\;size}{\underset{i=1}{\sum }}y_{i}.\log\hat{y_{i}}$$ (9)

### 2.3 Baseline methods

To test the performance of our method with other methods on annotating cell types of single-cell transcriptomics data, we compare TransCluster with the following baseline methods. Because our model is a deep learning method, which is a supervised learning method, in order to have more convincing experimental results, all the baseline methods we selected are supervised learning methods.

1. scDeepsort (Shao et al., 2021) is a graph-based method for single-cell category prediction. To construct the weighted cell-gene graph, cells and genes were both treated as graph nodes and the gene expression for each cell was regarded as the weighted edge between cells and genes.
2. SingleR (Aran et al., 2019) is an R package for automated cell type annotation of single cell RNA-seq sequencing (scRNA-seq) data, using a reference transcriptome dataset of pure cell types to independently infer the likely cell type of each cell.
3. ACTINN (Ma and Pellegrini, 2020) is a method for automatic cell class recognition using neural networks, which uses a neural network with three hidden layers trained on a dataset with predefined cell types and predicts the cell types of other datasets based on the trained parameters.
4. singleCellNet (Tan and Cahan, 2019) is able to classify cells across species based on the processed gene expression matrix.
5. scMap_cell (Kiselev et al., 2018) takes the cells in the query dataset as the nearest neighbors of the reference data, and the nearest neighbor cells in the reference dataset are most similar to the cells in the query dataset.

### 2.4 Metrics

We chose five metrics to evaluate the performance of the model, including accuracy, $f_{1-score}$ , precision, recall and matthews correlation coefficient (MCC). Since we are solving a multi-classification problem with unbalanced data for each category, we choose macro precision, macro recall and macro $f_{1-score}$ . These metrics have different focuses. Accuracy focuses on assessing the model’s ability to correctly classify samples, while macro $f_{1-score}$ focuses on assessing the sensitivity of the model. Macro precision addresses the question of how many of the samples that the model predicts as positive classes are predicted correctly, macro recall addresses the question of how many of the samples that the model predicts out of all positive classes. MCC focuses on the prediction of model classification performance in unbalanced datasets. We calculate accuracy, macro precision, macro recall, MCC and macro $f_{1-score}$ respectively by the following equations. Where TP, FP, FN and TN are short for the true positives, the false positives, the false negatives and the true negatives respectively (Shao et al., 2021). TP is a positive sample predicted by the model as a positive class. TN is the negative sample predicted as the negative class. FP is the negative sample predicted as positive class. FN is the positive sample predicted as the negative class.

$$Accuracy=\frac{TP+TN}{TP+TN+FP+FN}$$ (10)

$$Macro\;Precision=\frac{1}{l}\overset{l}{\underset{i=1}{\sum }}\frac{{TP}_{i}}{{TP}_{i}+{FP}_{i}}$$ (11)

$$Macro\;Recall=\frac{1}{l}\overset{l}{\underset{i=1}{\sum }}\frac{{TP}_{i}}{{TP}_{i}+{FN}_{i}}$$ (12)

$$MCC=\frac{TP*TN-FP*FN}{\sqrt{\left(TP+FP\right)*\left(TP+FN\right)*\left(TN+FP\right)*\left(TN+FN\right)}}$$ (13)

$$Macro\;F1=\frac{2\times Macro\;Precision\times Macro\;Recall}{Macro\;Precision+Macro\;Recall}$$ (14)

## 3 Results

### 3.1 Performance comparison of TransCluster with other methods on external test datasets

We compared the results of TransCluster with five baseline methods on tissues of lung, kidney and blood in Shao dataset (Shao et al., 2021). The five baseline methods included scDeepSort (Shao et al., 2021), SingleR (Aran et al., 2019), ACTINN (Ma and Pellegrini, 2020), singleCellNet (Tan and Cahan, 2019) and scMap_cell (Kiselev et al., 2018). In the datasets of different tissues, all cells from a specific tissue in the Shao dataset were selected as the training set, and test cells from the same tissues were used for cell type prediction. The processing of the datasets is described in detail in the Materials and Methods section. The experimental results of the baseline approach (Shao et al., 2021) are taken from the references, and the training and test sets used for all experiments are identical. The final results are shown in Table 1.

**TABLE 1:** Performance comparison of TransCluster with existing methods on different datasets. The bolded part is the best performance case.

Tissue model	Lung			Blood			Kidney		
	ACC	$F_{1-score}$	MCC	ACC	$F_{1-score}$	MCC	ACC	$F_{1-score}$	MCC
TransCluster	**0.7637**	**0.5942**	**0.6545**	**0.9429**	**0.8224**	**0.9050**	**0.9274**	**0.6804**	**0.8512**
scDeepSort	0.6622	0.5921	0.5990	0.9283	0.7993	0.7855	0.9173	0.6044	0.5402
SingleR	0.6150	0.5905	0.5923	0.6128	0.5135	0.4902	0.3155	0.3730	0.2934
ACTINN	0.7346	0.5763	0.5809	0.8327	0.6074	0.5911	0.7682	0.5536	0.4264
singleCellNet	0.7032	0.5115	0.4983	0.9152	0.8082	0.7812	0.7200	0.5203	0.3848
scMap_cell	0.3428	0.0424	0.2448	0.6115	0.3323	0.2899	0.0093	0	0.0348

Generally, from Table 1, it can be seen that TransCluster can predict cell classes in external test datasets after training in the training set, and the accuracy (ACC), $f_{1-score}$ and matthews correlation coefficient (MCC) are higher than other models. As shown in Table 1, in the lung, blood and kidney datasets, the best performance is found in the blood dataset with an ACC of 0.9429, $f_{1-score}$ of 0.8224, and MCC of 0.9050. In comparison, the performance in the lung dataset is poorer with an ACC of 0.7637, $f_{1-score}$ of 0.5942, and MCC of 0.6545, due to the fact that the lung dataset has more cell types and is more difficult to perform cell class identification. This is sufficient to demonstrate that our proposed model trained on a cellular dataset of a specific tissue and predicted cell type on another dataset of the same tissue. Since the training and test sets belong to different datasets of the same tissue, the gene classes of both are somewhat different and the results of feature learning are poor, resulting in generally a slightly lower accuracy than that of experiments performed on the same dataset.

### 3.2 Performance of TransCluster on different tissues

**FIGURE 2:**
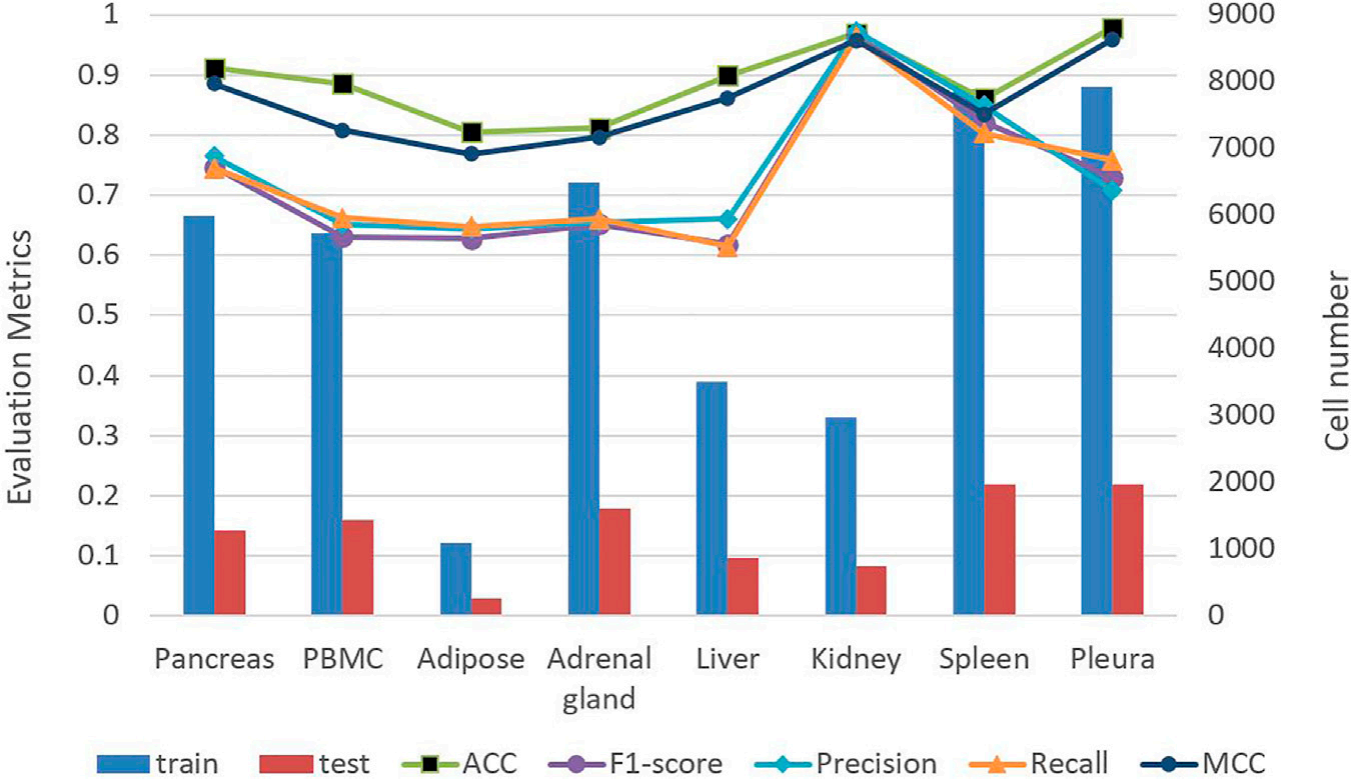
Performance of TransCluster on different tissues.

To demonstrate the universality of TransCluster, we used different data of human tissues from the Shao dataset (Shao et al., 2021) to measure the prediction results. We split each dataset into an 8:2 of the training set and test set, and take the average of five experiments as the final result. Part of the experimental results are shown in Figure 2. In which, train denotes the amount of data in the training set and test denotes the amount of data in the test set. As shown in Figure 2, we can easily find that the model has the highest ACC and MCC in the Pleura dataset with 0.9782 and 0.9595, respectively. The lowest ACC and MCC were obtained in the fat dataset with 0.8051 and 0.7684. The reason for this situation may be that the data volume of the Adipose dataset is too small, resulting in incomplete feature learning, while the Pleura dataset has a large enough data volume and a relatively small number of cell classes. Overall, it seems that TransCluster achieves more than 80% accuracy in several different human tissue datasets, which demonstrates the applicability of our model. Meanwhile, the higher values of ACC and MCC and the generally lower values of precision and recall are due to the very unequal distribution of the categories in the used dataset. For multi-categorization, ACC and MCC are more convincing indicators, and the performance of ACC and MCC is sufficient to illustrate the goodness of our model.

### 3.3 Sensitivity analysis

#### 3.3.1 Ablation experiments

**FIGURE 3:**
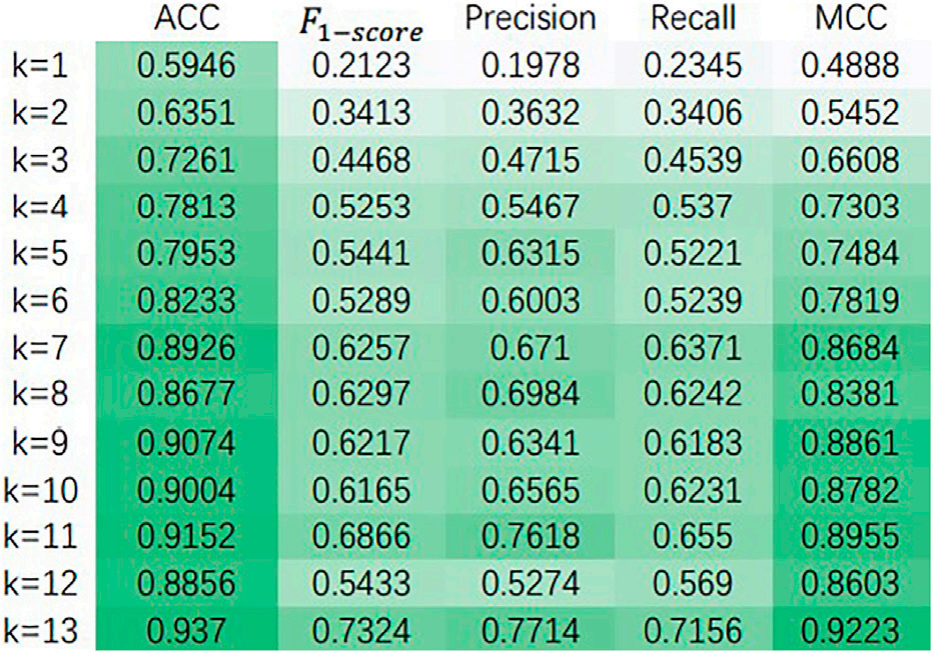
The effect of dimensionality reduction on the model.

For choosing the hyperparameters that make our model own the best performance, we did some sensitivity analysis experiments on the Baron dataset (Shao et al., 2021). First, in Table 2, we discuss the variation of various performance parameters of the model with or without decoders at different numbers of attentional heads. Meanwhile, we discuss the performance of the model under different dimensionality reduction, and the experimental results are shown in Figure 3.

**TABLE 2:** Performance of the model with or without the decoder section under different number of attention heads on Baron dataset. The bolded part is the best performance case.

Head-num	TransCluster with decoder					TransCluster without decoder				
	ACC	$F_{1-score}$	Precision	Recall	MCC	ACC	$F_{1-score}$	Precision	Recall	MCC
1	0.9105	0.7110	0.7217	0.7058	0.9007	0.9292	0.6787	0.7260	0.6655	0.9157
2	0.9152	0.6582	0.6658	0.6544	0.8975	0.9183	0.7172	0.8372	0.7037	0.8978
3	0.9012	0.6328	0.6443	0.6351	0.8647	0.9354	0.7182	0.7408	0.7098	0.9189
4	0.9144	0.6740	0.6920	0.6673	0.9012	0.9191	0.6168	0.6789	0.6138	0.8896
5	**0.9370**	**0.7324**	**0.7714**	**0.7156**	**0.9223**	**0.9354**	**0.7855**	**0.8001**	**0.7817**	**0.9201**
6	0.9268	0.7405	0.7661	0.7262	0.9056	0.9230	0.7345	0.7717	0.7146	0.8989
7	0.9307	0.6519	0.6573	0.6565	0.9136	0.9331	0.6907	0.7305	0.6854	0.9028
8	0.9160	0.6947	0.7218	0.6821	0.8549	0.9230	0.6734	0.6914	0.6637	0.8974
9	0.9160	0.6694	0.6882	0.6632	0.8561	0.9245	0.7192	0.7864	0.6873	0.8987
10	0.9160	0.6619	0.6888	0.6485	0.8354	0.9261	0.7307	0.7647	0.7252	0.9072

It is found that the performance of our model has very small fluctuation as the number of heads increases in Table 2. This means that for our model, too many attention heads have not made the model work better. The model performs well in both cases with or without the decoder part. And in both cases, the best result is achieved when head equals five. The highest ACC, $f_{1-score}$ , precision, recall and MCC of TransCluster with decoder are 0.9370, 0.7324, 0.7714, 0.7156 and 0.9223, respectively. The highest ACC, $f_{1-score}$ , precision, recall and MCC of TransCluster without decoder are 0.9354, 0.7855, 0.8001, 0.7817 and 0.9201. Therefore, our model is chosen to have the decoder part and the number of attention heads is chosen to be five.

Figure 3 shows the variation of ACC, $f_{1-score}$ , precision, recall and MCC of the model with different number of dimensionality reduction, and k in the figure indicates the number of dimensionality reduction. From Figure 3, It can be found that the accuracy of the model generally shows an increasing trend as the number of dimensionality reduction increases. Taking the Baron dataset (Shao et al., 2021) as an example, the accuracy of the model is highest when using LDA dimensionality reduction and keeping the number of features as 13, i.e., the number of cell categories in the dataset minus 1. This pattern was also found by experiments on other datasets, so the number of downscaled retained features was chosen by subtracting 1 from the number of cell categories.

#### 3.3.2 Availability of the main part of the TransCluster

**FIGURE 4:**
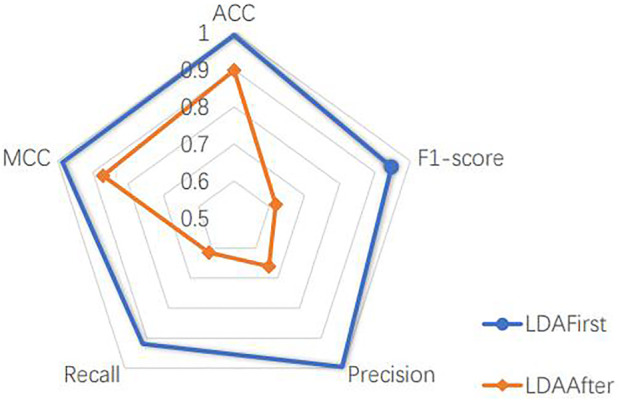
LDA’s position experiment.

The model includes a linear discriminant analysis (LDA) dimensionality reduction part, which is placed after dividing the dataset. In order to determine the location of the LDA, we experimented with the method of dimensionality reduction before dividing the dataset. Since the dimensionality reduction better represents the features of different cell classes, as shown in Figure 4, taking the liver dataset as an example, the performance of the model is better to reduce the dimension of the whole dataset and then split it than to reverse the order of two operations. This is because the LDA dimensionality reduction process uses cell labels as a reference, which makes its selection of the main features more accurate. However, the actual cells to be predicted have no prior knowledge of the labels, therefore, it is more reasonable to choose the way of dividing the dataset first. This experiment is sufficient to justify the LDA location and also reveals the usability of the main part of the model.

#### 3.3.3 Visual analysis

**FIGURE 5:**
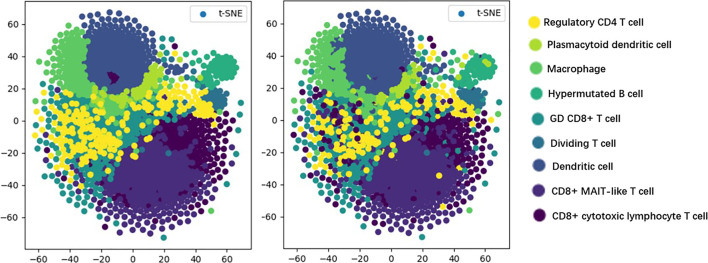
Visualization results.

T-distributed stochastic neighbor embedding (t-SNE) (Maaten and Hinton, 2008) is a machine learning algorithm used for dimensionality reduction, which can visualize high-dimensional data, so that we have an intuitive understanding of the distribution of data. As shown in Figure 5, we visualize the prediction results of the model by the t-SNE method in order to discover the testing effect of the model more intuitively.

In Figure 5, the visualization of real cell classes is shown on the left, and the distribution of cell classes predicted by TransCluster is shown on the right (taking the optimal prediction result of Spleen dataset as an example). We can know that the vast majority of cells are predicted accurately except for very few cells.

#### 3.3.4 Confusion matrix

**FIGURE 6:**
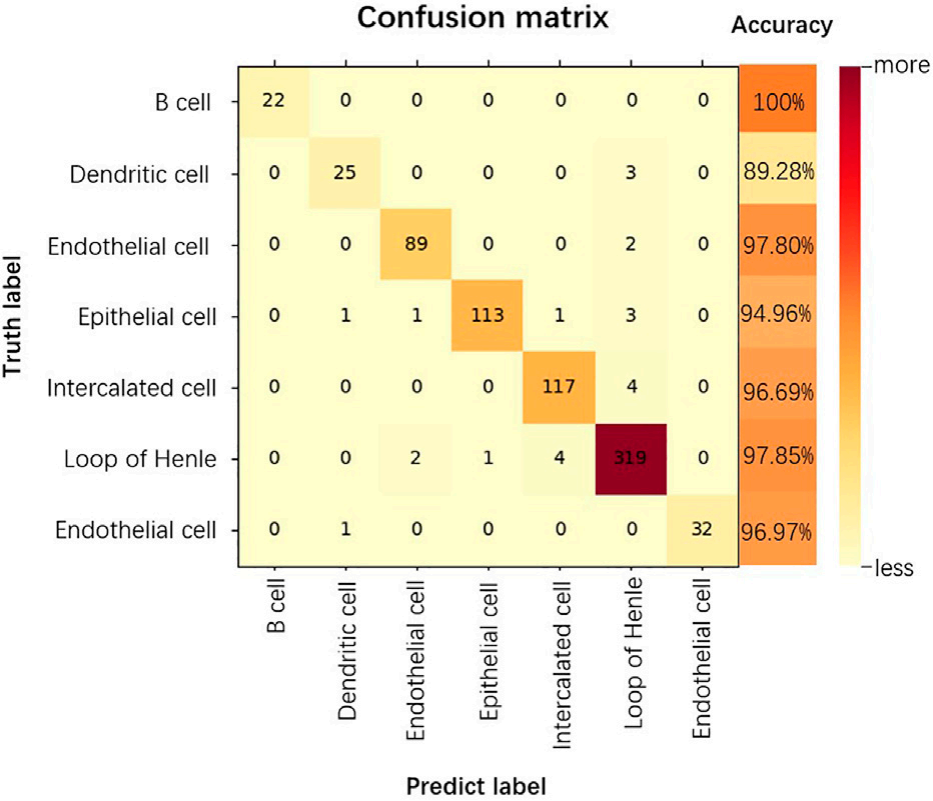
Confusion matrix for the classification results of the Kidney dataset.

The confusion matrix is a summary of the predictions for a classification problem. The number of correct and incorrect predictions is summarized using count values and broken down by each category, which is the key to the confusion matrix (Görtler et al., 2022). The confusion matrix shows which part of the classification model is confused when making predictions, providing insight not only into the errors made by the classification model, but more importantly, the types of errors that occur, overcoming the limitations associated with using classification accuracy alone (Li et al., 2022). As shown in Figure 6, we show the confusion matrix of the classification results for the kidney dataset.

As can be seen from Figure 6, for the kidney dataset, of the 740 predicted data for the 7 cell categories, the category that could all be accurately predicted is B cell, 10.71% of Dendritic cells and 2.19% of Endothelial cells are incorrectly categorized as Loop of Henle. 94.96% of Epithelial cells are accurately predicted, the probability of being incorrectly predicted as Dendritic cells, Endothelial cells, Intercalated cells are all 0.84%, the probability of being predicted as Loop of Henle is 2.52%. The accuracy of prediction of Loop of Henle is 97.85%, and that of Endothelial cell is 96.97%. 3.30% of Intercalated cells are incorrectly classified as Loop of Henle. thus, the prediction accuracy of B cell is the highest, and Loop of Henle caused the most interference to the model prediction.

## 4 Discussion

In this study, we proposed a single-cell category prediction model, TransCluster, which adopts a unique dimensionality reduction approach and feature extraction method. Unlike other methods, TransCluster begins with gene expression matrix processing by LDA’s dimensionality reduction method to ensure that the features being learned are more targeted. At the same time, the number of parameters is greatly reduced, making the model run much faster than other baseline methods. The modified Transformer is used for feature information extraction, which makes the extracted features closer to the target data and more effective than other methods.

To our knowledge, which is the first application of the Transformer module to the field of single-cell category prediction. Extensive experiments in the human scRNA-seq dataset have shown that our model is able to accurately predict the majority of cells in multiple human tissues. Comparison with other models reveals that our model can achieve state-of-the-art prediction performance, which demonstrates the feasibility of the Transformer module in cell classification tasks.

There are some aspects of our approach that could be improved in the future. Due to the rapid development of graph neural networks (Wu et al., 2021), models with constructed cellular relationship graphs are starting to emerge in the field of cell type prediction. We will use graphs to improve the cell type identification pipeline. We expect that over time, more cell types from larger maps should be used to train more comprehensive neural networks. In the future, we will apply single-cell datasets containing more data information to single-cell category prediction.

## Data Availability

The source code is available at https://github.com/Danica123/TransCluster.git and all datasets are publicly available at https://github.com/Danica123/TransCluster/releases/tag/Dataset.
